# A Data‐Driven Epigenetic Characterization of Morning Fatigue Severity in Oncology Patients Receiving Chemotherapy: Associations With Epigenetic Age Acceleration, Blood Cell Types, and Expression‐Associated Methylation

**DOI:** 10.1002/cam4.71067

**Published:** 2025-07-25

**Authors:** Caroline Le, Maureen Lewis, Carolyn S. Harris, Liam Berger, Esther Chavez‐Iglesias, Lisa Morse, Anatol Sucher, Ritu Roy, Adam Olshen, Marilyn J. Hammer, Steve Paul, Margaret Wallhagen, Raymond Chan, Michael Sayer, Sue Yom, Nam‐Woo Cho, Alexandre Chan, Jon Levine, Anand Dhruva, Christine Miaskowski, Yvette P. Conley, Kord M. Kober

**Affiliations:** ^1^ Department of Physiological Nursing University of California San Francisco San Francisco California USA; ^2^ University of California San Francisco Helen Diller Family Comprehensive Cancer Center San Francisco California USA; ^3^ University of Pittsburgh School of Nursing Pittsburgh Pennsylvania USA; ^4^ Department of Epidemiology and Biostatistics University of California San Francisco San Francisco California USA; ^5^ Division of Population Sciences Dana‐Farber Cancer Institute, Nursing and Patient Care Services/Medical Oncology Boston Massachusetts USA; ^6^ College of Nursing and Health Sciences, Caring Future Institutes Flinders University Adelaide South Australia Australia; ^7^ University of California Irvine School of Pharmacy Irvine California USA; ^8^ Department of Radiation Oncology University of California San Francisco San Francisco California USA; ^9^ School of Dentistry University of California San Francisco San Francisco California USA; ^10^ University of California San Francisco Osher Center for Integrative Health San Francisco California USA; ^11^ Department of Human Genetics University of Pittsburgh Graduate School of Public Health Pittsburgh Pennsylvania USA

## Abstract

**Background:**

Moderate‐to‐severe fatigue commonly occurs in patients with cancer. Given the numerous roles that epigenetic processes may play in the development and severity of fatigue, the purposes of this study were to (1) use a data‐driven discovery approach to evaluate for mechanisms underlying morning fatigue in a group of oncology patients receiving chemotherapy and (2) identify common biological mechanisms associated with morning fatigue severity across these independent epigenetic evaluations.

**Methods:**

Patients completed questionnaires during the week prior to their chemotherapy treatment. Severity of morning fatigue was evaluated using the Lee Fatigue Scale. Associations between morning fatigue severity and epigenetic aging acceleration (EAA), immune cell type compositions, and differential methylation of expression‐associated loci (eCpGs) in distal regions (i.e., upstream of a gene on the same chromosome) were evaluated. These results were then evaluated for common biological mechanisms.

**Results:**

High morning fatigue was associated with older epigenetic age, positive EAA, and higher levels of EAA. Patients of the “Fast ager” type were more likely to have high morning fatigue. Higher morning fatigue was associated with lower (CD4 memory, CD8 memory, and NK) and higher (neutrophil and T regulatory) estimated proportions of cell types. Morning fatigue severity was associated with one differentially methylated distal region containing five eCpGs mapping to three genes (i.e., CILP, ONECUT1, SLCO3A1). Preliminary support was found for the role of Inflammaging as a common biological mechanism for morning fatigue.

**Conclusions:**

This study provides an epigenetic characterization of morning fatigue in patients receiving chemotherapy. The findings suggest that biological aging, gene regulatory, and inflammatory processes may contribute to morning fatigue and provide future targets for therapeutic interventions.

## Graphical abstract

Fatigue is commonly associated with cancer and its treatments and has a negative impact on patients' ability to tolerate treatments and on their quality of life. This epigenetic characterization of morning fatigue in patients receiving chemotherapy finds biological aging, gene regulatory, and inflammatory processes associated with morning fatigue.

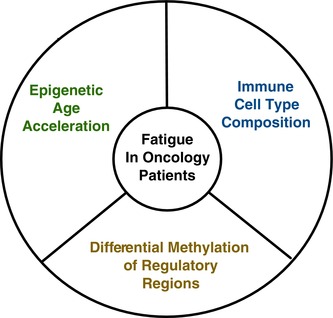

## 1 Introduction

Fatigue is the most common symptom associated with cancer and its treatments [1, 2]. It has a negative impact on patients' ability to tolerate treatments and on their quality of life (QOL) [3]. Fatigue severity demonstrates a large amount of interindividual variability based on multiple demographic, clinical, behavioral, and biological characteristics [4, 5, 6, 7, 8]. Given its high occurrence and negative impact, effective treatments are urgently needed for this devastating symptom [9]. One of the major knowledge gaps for the effective treatment of fatigue is the lack of knowledge of its underlying mechanisms [4, 8, 9, 10, 11, 12].

**FIGURE 1:**
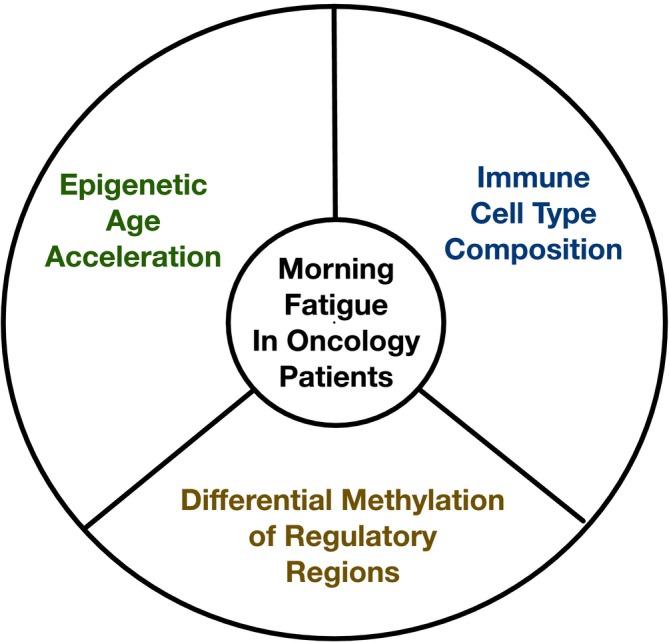
Conceptual image of the epigenetic characterization of morning fatigue in oncology patients receiving chemotherapy using a data‐driven discovery approach.

Epigenetic processes serve as an interface between the environment and the individual [13]. Epigenetic mechanisms are involved in numerous biological processes that may be associated with fatigue, including biological aging, immune cell type function, and gene regulation (Figure 1) [14]. Although epigenetic modifications may contribute to the development and severity of fatigue [15, 16, 17, 18], the majority of previous ‘omics studies of fatigue have focused on genetics or gene expression [5]. To better understand its underlying mechanisms, there is a need for an epigenetic characterization of morning fatigue across these three epigenomic processes in oncology patients undergoing chemotherapy (CTX).

One of the challenges in investigating fatigue is that the etiology is multifactorial (reviewed in [19, 20]). To address some of the complexity, a data‐driven approach can be used to evaluate mechanisms of morning fatigue severity in patients receiving chemotherapy (CTX). Data‐driven analyses in biology utilize large amounts of data (e.g., ‘omics’) to generate useful predictions of biological processes [21]. In terms of symptoms in oncology patients receiving CTX, previous studies have used a data‐driven discovery approach using transcriptomic data to identify potential biological mechanisms associated with the severity of nausea [22], shortness of breath [23], and joint profiles of cancer‐related cognitive impairment and anxiety [24]. These data‐driven studies provide hypotheses that guide subsequent studies testing prognostication or therapeutic intervention. Studies using data‐driven approaches with other omics data (i.e., epigenetics) are needed to further understand the mechanisms for fatigue in oncology patients. In the current study, independent data‐driven analyses for three biological processes (i.e., biological aging, cell‐type function, and gene regulation) are evaluated to identify mechanisms associated with morning fatigue severity. These results are then reviewed across analyses to identify potential common mechanisms and directions for future research on underlying mechanisms.

In terms of aging, higher fatigue is associated with younger chronological age in oncology patients receiving chemotherapy [25, 26]. One hypothesis for this association is age‐related changes in the immune system [4], where “biologically” older (i.e., the measure of the functional capability of a person or organ and how it changes with age [27, 28, 29]), rather than chronologically older, patients would be at a greater risk for more severe fatigue profiles. Numerous measures are available to quantify biological age using epigenetic markers [30], which can be used to evaluate epigenetic age acceleration (EAA; i.e., the difference between biological versus chronological age) [31]. In a recent longitudinal study of 133 patients with head and neck cancer who were receiving curative‐intent radiation therapy, patients who reported severe fatigue had higher changes in EAA than patients who did not [15]. In addition, elevated inflammatory cytokines (e.g., CRP, IL‐6, IL‐1ra, IL‐10, sTNFR2) were associated with higher levels of EAA changes. In another longitudinal study of 72 patients with breast cancer who were receiving chemotherapy, increased EAA was associated with increased fatigue over the course of treatment [32]. Given the strength of these initial reports, additional studies are needed to evaluate for EAA changes associated with morning fatigue severity in additional patient groups, larger sample sizes, treatment types, and additional cancer diagnoses.

In terms of immune cell composition and function in relation to fatigue severity, a recent study of 717 oncology patients with various types of cancer receiving chemotherapy [33] found that morning fatigue severity was associated with gene expression perturbations in inflammatory pathways, many of them specific to immune cell types found in peripheral blood (i.e., Neutrophile extracellular trap formation, Th17 cell differentiation, Natural killer cell‐mediated cytotoxicity, and B‐cell receptor signaling pathway). While the mechanisms underlying fatigue in patients with cancer are hypothesized to be multifactorial [19, 20], the majority of the evidence supports inflammatory mechanisms [10, 16, 34, 35, 36, 37]. Given the impact of epigenetics on immune cell differentiation [38] and cell states [39], and the heterogeneity of roles of different peripheral blood immune cell types and their potential role in disease and complex traits [40], studies are needed to evaluate immune cell types that are associated with fatigue severity in oncology patients receiving chemotherapy.

Finally, epigenetic gene regulatory mechanisms may contribute to fatigue. Gene expression is managed through various processes, including transcription, post‐transcriptional modifications, and epigenetics [41]. One epigenetic process, DNA methylation [42], occurs primarily at the cytosine base of the molecule adjacent to guanine (i.e., CpG site). Gene expression can be associated with both decreased [43] and increased [44] methylation in regulatory regions, as well as decreased methylation within the gene [45]. In a study that evaluated the methylome of breast cancer patients prior to chemotherapy [46], increases in the severity of fatigue were associated with decreased methylation of CpG sites. This decreased methylation was correlated with increased inflammatory markers prior to and 6 months after chemotherapy. In our previous studies that evaluated gene expression patterns in patients receiving chemotherapy, morning [33] and evening [16, 33, 36, 37] fatigue severity were associated with perturbations in pathways involved in inflammation, neurotransmitter regulation, circadian rhythms, renal function, and energy metabolism. To better understand the regulatory processes underlying gene expression perturbations associated with morning fatigue severity, an evaluation of differential methylation of expression‐associated CpG (eCpG) loci in putative regulatory regions is needed.

**FIGURE 2:**
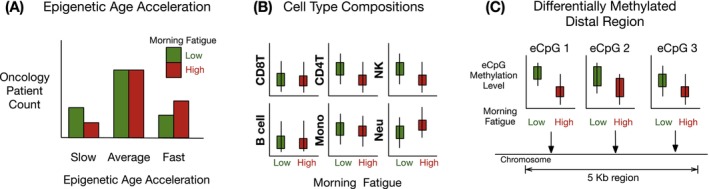
Overview of the study design. A pictorial depiction of the independent epigenetic characterizations of morning fatigue in patients receiving chemotherapy. The images depicted are for illustration of the design and do not present real results. (A) Tests for an association between epigenetic age acceleration and morning fatigue severity. (B) Evaluation for associations between morning fatigue and epigenetic estimates of cell type composition. (C) Tests for differentially methylated regions of expression associated with CpG loci (eCpGs) associated with morning fatigue.

Given the numerous roles that epigenetic processes may play in the development and severity of fatigue, this study uses a data‐driven discovery approach to further the understanding of the mechanisms underlying morning fatigue in a group of oncology patients receiving chemotherapy. Given that previous research demonstrated diurnal variations in fatigue severity [25, 26, 47], and the scope of the proposed analyses, this study focused on morning fatigue severity. The first purpose of this study was to use a data‐integrated [48, 49, 50] systems biology [51] approach to perform an epigenetic characterization of morning fatigue in oncology patients receiving chemotherapy through the evaluations for associations between morning fatigue severity and (A) EAA, (B) immune cell type compositions, and (C) differential methylation of distal regions (i.e., upstream of a gene on the same chromosome) of eCpG loci (Figure 2). The second purpose of this study was to identify common biological mechanisms associated with morning fatigue severity across these independent epigenetic evaluations and identify potential biological processes for further research.

## 2 Methods

### 2.1 Patients and Settings

This analysis is part of a larger study that evaluated the symptom experience of oncology outpatients receiving chemotherapy [52]. Eligible patients were ≥ 18 years of age; had a diagnosis of breast, lung, gastrointestinal, or gynecologic cancer; had received chemotherapy within the preceding 4 weeks; were scheduled to receive at least two additional cycles of chemotherapy; were able to read, write, and understand English; and gave written informed consent. Patients were recruited from two Comprehensive Cancer Centers, one Veterans Affairs hospital, and four community‐based oncology programs.

### 2.2 Study Procedures

The study was approved by the Committee on Human Research at the University of California San Francisco and the Institutional Review Board at each of the study sites. Of the 2234 patients approached, 1343 consented to participate (60.1% response rate). The major reason for refusal was being overwhelmed with their cancer treatment. Eligible patients were approached in the infusion unit during their first or second cycle of chemotherapy by a member of the research team to discuss study participation and obtain written informed consent. Data from the enrollment assessment (i.e., symptoms in the week prior to the patient's second or third cycle of chemotherapy) were used in this analysis. At enrollment, patients provided a blood sample for the deoxyribonucleic acid (DNA) methylation (*n* = 700) and ribonucleic acid (RNA) (*n* = 115) analyses.

### 2.3 Instruments

#### 2.3.1 Patient Demographic and Clinical Characteristics

Patients completed a demographic questionnaire, Karnofsky Performance Status (KPS) scale [53], Self‐Administered Comorbidity Questionnaire (SCQ) [54], and Alcohol Use Disorders Identification Test (AUDIT) [55]. Toxicity and emetogenicity of the chemotherapy regimen were evaluated using the MAX2 index [56] and published guidelines [57], respectively. Medical records were reviewed for disease and treatment information. Missing data for demographic and clinical characteristics were imputed by the k‐nearest‐neighbors method, as described previously [33].

#### 2.3.2 Lee Fatigue Scale

The 18‐item Lee Fatigue Scale (LFS) was used to assess physical fatigue and energy [58]. Each item was rated on a 0–10 numeric rating scale. Mean scores were calculated for the 13 fatigue items. Higher scores indicate greater fatigue severity. Patients rated each item based on how they felt within 30 min of awakening (i.e., morning fatigue) and prior to going to bed (i.e., evening fatigue). This study will focus on morning fatigue. Mean scores for morning fatigue were used in this analysis.

### 2.4 Acquisition and Processing of DNA Methylation Data

DNA methylation analyses are described in detail elsewhere [59]. In brief, total DNA isolated from peripheral blood was used as a template to quantify DNA methylation in two separate batches. DNA methylation for 115 patients was quantified using the Infinium HumanMethylation450 BeadChip (Illumina Inc., San Diego, CA) and was used for the eQTM mapping analysis. DNA methylation for 585 patients was quantified using the Infinium MethylationEPIC BeadChip (Illumina Inc., San Diego, CA) and was used for the epigenetic age estimates, estimates of cell‐type compositions, and differential methylation analyses. DNA methylation data are available in the NCBI database of Genotypes and Phenotypes (dbGaP) under Study ID phs003863. Details of the expression quantitative trait methylation (eQTM) mapping analysis are described below. Corrections for probe type, balance correction, background correction, and quantile normalization were performed using the minfi package (version 1.40.0) [60, 61]. Methylation values were quantified as M‐values for differential methylation analyses [62].

### 2.5 Analysis of Demographic and Clinical Characteristics

Data were analyzed using R (version 4.1, https://www.R‐project.org/). Patients were classified into low (< 3.2) and high (≥ 3.2) morning fatigue groups using the clinically meaningful cutoff score [63]. Differences in demographic and clinical characteristics between the patients in the low and high fatigue groups were evaluated using parametric and nonparametric tests. To identify covariates to include in the differential methylation analyses, significant characteristics (*p* < 0.05) were entered into a multiple logistic regression analysis. For the final model, variables were selected using a backward stepwise logistic regression approach based on the likelihood ratio test. The area under the curve of the receiver operating characteristic curve was used to evaluate the overall adequacy of the regression model [64].

### 2.6 Epigenetic Estimates of Biological Age

Epigenetic age was estimated using the DNAm PhenoAge method [65]. Epigenetic age was estimated for patients in this study using the model and coefficient values provided [65]. EAA was calculated as chronological age subtracted from epigenetic age [65, 66, 67, 68]. An association between morning fatigue severity and EAA was evaluated using Welch's *t*‐test (Figure 2A), and significance was assessed at *p* < 0.05. Guided by Levine (2018) [65], three types of EAA were created (i.e., “Slow ager” [EAA < −10], “Average ager” [−10 ≤ EAA ≤ 10], and “Fast ager” [EAA > 10]). Associations between the EAA type and morning fatigue group were evaluated using Pearson's chi‐squared test for count data. The significance of the pairwise differences was assessed at a family‐wise error rate of < 0.05.

### 2.7 Epigenetic Estimates of Cell‐Type Composition

To provide a detailed immune profile from peripheral blood, blood cell types [69] were estimated using the *estimateCellCounts2()* function in the FlowSorted.BloodExtended.EPIC R package (version 1.1.2) [70, 71]. Cell‐type deconvolution was performed using the IDOL library (i.e., IDOLOptimizedCpGsBloodExtended) for 12 cell types: basophil, B memory, B naïve, cluster of differentiation 8 (CD8) memory T, CD8 naïve T, CD4 memory T, CD4 naïve T, eosinophil, monocyte, natural killer (NK), neutrophil, and T regulatory [71, 72]. Associations between the morning fatigue severity groups and estimations of cell‐type compositions were evaluated using Welch's *t*‐test (Figure 2B). The significance of these tests was assessed at *p* < 0.05.

### 2.8 Identification of Expression‐Associated CpG Loci (eCpGs)

There is growing interest in understanding the role of DNA methylation in gene expression by evaluating the relationships between gene expression levels and the methylation status of nearby genes. Recent studies have evaluated associations with gene expression levels and the methylation status of regions further from the gene (e.g., putative enhancer sites) or even on different chromosomes [73]. Given these methylation‐expression associations vary with the distance from the promoter [74], as well as between individuals and across tissues [75], studies are needed that identify eCpG sites from patient samples that are closely related to the study of interest (i.e., blood from oncology patients receiving chemotherapy) and from regulatory regions distant from a gene. To identify eCpG loci, eQTM mapping analysis was performed using an independent group of 115 oncology patients receiving chemotherapy for which both gene expression and methylation microarray data were available. Total RNA isolated from peripheral blood was quantified for 115 patients using the HumanHT‐12 v4.0 Expression BeadChip (Illumina, San Diego, CA) microarray. The eQTM analysis was performed using the Torch‐eCpG tool [76] (https://github.com/kordk/torch‐ecpg). The DNA methylation and gene expression analysis, sample characteristics, and the eQTM analysis are described in detail in (File S1).

### 2.9 Evaluation for Differential Methylation of Regions Associated With Morning Fatigue Severity

In the evaluation group of patients (*n* = 584 total, *n* = 224 High and *n* = 360 Low morning fatigue), methylation was measured at 794,441 loci following our previously published quality control and data processing methods [59]. Of these loci, only those identified as Distal eCpGs (i.e., upstream of a gene on the same chromosome) were evaluated for differential expression in this analysis. Methylation levels were quantified as the M‐score. Surrogate variable analysis using the Leek method (R package version 3.4.0) [77] was used to estimate surrogate variables for technical and nontechnical variations that contributed to heterogeneity in the sample that were not due to fatigue or significant demographic or clinical phenotype characteristics. The final differential methylation regression model included surrogate variables and all of the demographic and clinical variables retained in the final backward stepwise regression model (Table 2) as covariates.

To evaluate for associations between morning fatigue group membership and methylation levels in the distal putative regulatory regions [78] of genes (Figure 2C), tests for differential methylation of individual probes were done using a generalized linear model implemented in the limma R package using the “ls” method (version 3.48.3) [79]. Methylation regions were defined as those having ≥ 2 eCpGs in a sliding window of 5000 bases. Differentially methylated regions (DMRs) were determined using Fisher's combined probability test, which combined the individual differential methylation tests for a region [80]. Using this approach, all tests for DMRs within the distal putative regulatory regions of a given gene were represented by a single, uncorrected *p* value. The significance of these DMRs was assessed using a false discovery rate (FDR) cutoff of 0.05. To characterize the potential functional roles of a significant DMR, the direction of expression associated with methylation levels was identified (i.e., eCpGs). In addition, evidence of regulatory elements in the region surrounding the DMR was provided using annotation data from the Encyclopedia of DNA Elements (ENCODE) [81] obtained from the hg19 assembly on the University of California Santa Cruz Genome Browser [82].

## 3 Results

### 3.1 Patient Demographic and Clinical Characteristics of Morning Fatigue Groups

Differences in patient demographic and clinical characteristics between the morning fatigue groups are provided in Table 1. Six characteristics were retained in the final logistic regression model and used as covariates in the differential methylation analyses (Table 2). Compared to patients in the low group, patients in the high morning fatigue group were younger, less likely to be married or partnered, and less likely to exercise on a regular basis. In terms of clinical characteristics, compared with the low group, patients in the high group had a lower functional status, higher comorbidity burden, and were less likely to have a diagnosis of gastrointestinal cancer.

**TABLE 1:** Differences in demographic and clinical characteristics between patients with low and high morning fatigue.

Characteristic	Low morning fatigue 61.6% (*n* = 360)	High morning fatigue 38.4% (*n* = 224)	Statistics
	Mean (SD)	Mean (SD)	
Chronological age (years)	59.6 (11.7)	55.1 (12.5)	*t* = 4.29, *p* < 0.001
Education (years)	16.2 (3.0)	16.0 (2.9)	*t* = 0.79, *p* = 0.427
Body mass index (kg/m^2^)	25.8 (5.5)	26.4 (5.4)	*t* = −1.44, *p* = 0.150
KPS score	85.0 (11.3)	76.5 (12.1)	*t* = 8.48, *p* < 0.001
Number of comorbidities	2.2 (1.4)	2.5 (1.4)	*t* = −2.32, *p* = 0.021
SCQ score	4.8 (2.8)	5.8 (3.4)	*t* = −3.88, *p* < 0.001
AUDIT score	3.0 (1.8)	2.9 (1.9)	*t* = 0.58, *p* = 0.563
Time since diagnosis (years)	2.0 (4.3)	2.1 (4.6)	*U*, *p* = 0.118
Time since diagnosis (years, median)	0.40	0.42	
Number of prior cancer treatments	1.5 (1.5)	1.6 (1.5)	*t* = −1.16, *p* = 0.245
Number of metastatic sites including lymph node involvement	1.2 (1.2)	1.2 (1.2)	*t* = 0.09, *p* = 0.932
Number of metastatic sites excluding lymph node involvement	0.8 (1.0)	0.8 (1.0)	*t* = 0.20, *p* = 0.844
MAX2 score	0.2 (0.1)	0.2 (0.1)	*t* = −1.49, *p* = 0.138
Hemoglobin (g/dL)	11.6 (1.4)	11.3 (1.4)	*t* = 1.96, *p* = 0.051
Hematocrit (%)	34.7 (4.0)	34.1 (4.2)	*t* = 1.84, *p* = 0.066
LFS Morning Fatigue score at enrollment	1.3 (0.9)	5.1 (1.4)	*t* = −35.78, *p* < 0.001
LFS Evening Fatigue score at enrollment	4.3 (2.1)	6.3 (1.8)	*t* = −12.17, *p* < 0.001
	**%** **(** ***n*** **)**	**%** **(** ***n*** **)**	
Gender			FE, *p* = 0.002
Female	71.7 (258)	83.0 (186)	
Male	28.3 (102)	17.0 (38)	
Ethnicity			*X* ^2^ = 3.67, *p* = 0.299
White	71.9 (259)	76.3 (171)	
Black	8.1 (29)	5.8 (13)	
Asian or Pacific Islander	10.8 (39)	7.1 (16)	
Hispanic mixed or other	9.2 (33)	10.7 (24)	
Married or partnered (% yes)	69.7 (251)	59.8 (134)	FE, *p* = 0.015
Lives alone (% yes)	18.3 (66)	24.6 (55)	FE, *p* = 0.075
Childcare responsibilities (% yes)	17.2 (62)	27.2 (61)	FE, *p* = 0.005
Care of adult responsibilities (% yes)	5.8 (21)	7.1 (16)	FE, *p* = 0.601
Born prematurely (% yes)	5.6 (20)	5.4 (12)	FE, *p* = 1.000
Currently employed (% yes)	40.6 (146)	32.1 (72)	FE, *p* = 0.043
Income			
<$30,000	10.8 (39)	17.4 (39)	*U*, *p* = 0.108
<$30,000 to <$70,000	22.2 (80)	21.9 (49)	
$70,000 to <$100,000	21.1 (76)	18.8 (42)	
≥ $100,000	46.0 (166)	42.0 (94)	
Specific comorbidities (% yes)			
Heart disease	5.0 (18)	5.8 (13)	FE, *p* = 0.706
High blood pressure	33.3 (120)	25.0 (56)	FE, *p* = 0.033
Lung disease	11.4 (41)	12.5 (28)	FE, *p* = 0.694
Diabetes	6.9 (25)	9.8 (22)	FE, *p* = 0.215
Ulcer or stomach disease	5.6 (20)	4.0 (9)	FE, *p* = 0.441
Kidney disease	1.9 (7)	1.8 (4)	FE, *p* = 1.000
Liver disease	6.1 (22)	5.4 (12)	FE, *p* = 0.856
Anemia or blood disease	12.2 (44)	13.4 (30)	FE, *p* = 0.702
Depression	6.9 (25)	31.7 (71)	FE, *p* < 0.001
Osteoarthritis	11.7 (42)	9.8 (22)	FE, *p* = 0.586
Back pain	18.1 (65)	26.8 (60)	FE, *p* = 0.017
Rheumatoid arthritis	1.7 (6)	3.1 (7)	FE, *p* = 0.261
Exercise on a regular basis (% yes)	76.7 (276)	67.9 (152)	FE, *p* = 0.021
Smoking current or history of (% yes)	31.4 (113)	39.3 (88)	FE, *p* = 0.060
Cancer diagnosis			*χ* ^2^ = 9.03, *p* = 0.029
Breast	38.2 (138)	48.2 (108)	NS
Gastrointestinal	35.6 (128)	24.6 (55)	0 > 1
Gynecological	14.4 (52)	16.5 (37)	NS
Lung	11.7 (42)	10.7 (24)	NS
Type of prior cancer treatment			*χ* ^2^ = 1.43, *p* = 0.699
No prior treatment	28.8 (104)	24.6 (55)	
Only surgery, CTX, or RT	41.0 (148)	42.0 (94)	
Surgery & CTX, or surgery & RT, or CTX & RT	19.4 (70)	21.4 (48)	
Surgery & CTX & RT	10.8 (39)	12.1 (27)	
CTX cycle length			*χ* ^2^ = 3.67, *p* = 0.159
14 day cycle	44.4 (160)	41.5 (93)	
21 day cycle	47.8 (172)	54.0 (121)	
28 day cycle	7.8 (28)	4.5 (10)	
Emetogenicity of CTX			*χ* ^2^ = 1.51, *p* = 0.469
Minimal/low	18.9 (68)	21.0 (47)	
Moderate	62.2 (224)	57.1 (128)	
High	18.9 (68)	21.9 (49)	
Antiemetic regimens			*χ* ^2^ = 2.85, *p* = 0.415
None	7.8 (28)	6.7 (15)	
Steroid alone or SRA alone	20.0 (72)	25.4 (57)	
SRA and steroid	49.7 (179)	44.6 (100)	
NK‐1 receptor antagonist and two other antiemetics	22.5 (81)	23.2 (52)	

Abbreviations: *χ* ^2^, chi square; AUDIT, Alcohol Use Disorders Identification Test; CTX, chemotherapy; dL, deciliter; FE, Fisher's exact test; g, grams; kg, kilograms; KPS, Karnofsky Performance Status; LFS, Lee Fatigue Scale; m^2^, meter squared; NK‐1, neurokinin‐1; NS, not significant; RT, radiation therapy; SCQ, Self‐administered Comorbidity Questionnaire; SD, standard deviation; SRA, serotonin receptor antagonist; *U*, Mann–Whitney *U* test.

**TABLE 2:** Multiple logistic regression analysis for high morning fatigue group membership.

Oncology patients (*n* = 585; low MF 61.6%, *n* = 360, high MF 38.4%, *n* = 224)			
Predictors	Odds ratio	95% CI	*p*
Age (years)	0.97	0.95, 0.98	2.69 × 10^−5^
Karnofsky Performance Status score	0.95	0.93, 0.97	1.579 × 10^−9^
Self‐Administered Comorbidity Questionnaire score	1.08	1.01, 1.15	0.030
Married/partnered (yes)	0.64	0.43, 0.94	0.023
Exercise on a regular basis (yes)	0.72	0.47, 1.10	0.130
Cancer diagnosis			
Breast	1.00		
Gastrointestinal	0.51	0.33, 0.80	0.004
Gynecological	0.85	0.49, 1.47	0.558
Lung	0.71	0.36, 1.38	0.320

*Note:* Overall model fit: AUC of the ROC = 0.750.

Abbreviations: AUC, area under curve; CI, confidence interval; MF, morning fatigue; ROC, receiver operating characteristic.

### 3.2 Epigenetic Estimates of Biological Age and EAA Associated With Morning Fatigue Severity

**FIGURE 3:**
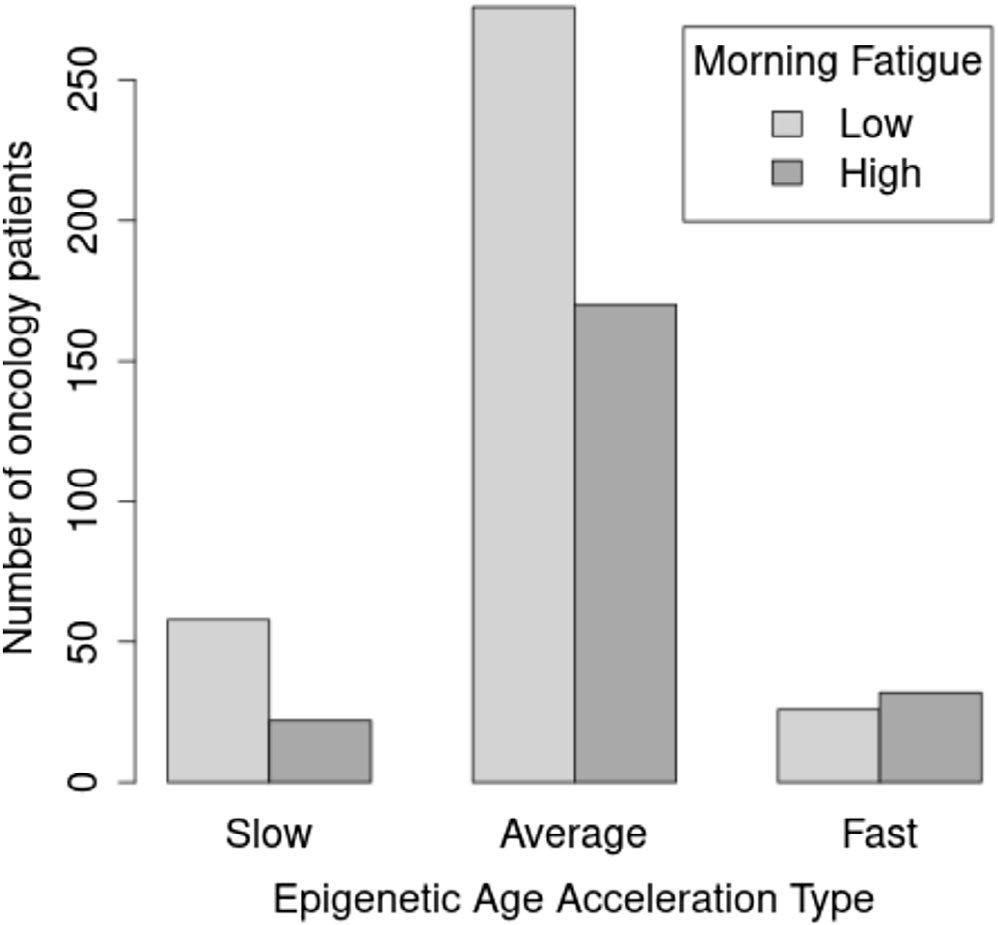
Morning fatigue severity and epigenetic age type in 585 patients receiving chemotherapy. Epigenetic age acceleration (EAA) aging types are defined as “Slow” (EAA < −10), “Average” (−10 ≤ EAA ≤ 10), and “Fast” (EAA > 10).

Estimates of epigenetic age were strongly correlated with chronological age (Pearson's *r* = 0.753, *p* value < 2.2 × 10^−16^). High morning fatigue was associated with younger epigenetic age, positive EAA, and higher levels of EAA (Table 3). In terms of EAA ager types, as compared to patients of the “Slow ager” type, patients of the “Faster ager” type were more likely to have high morning fatigue (Table 3, Figure 3).

**TABLE 3:** Differences in estimates of epigenetic age and epigenetic age acceleration between the morning fatigue severity groups.

Estimated characteristic	Low morning fatigue 61.6% (*n* = 360)	High morning fatigue 38.3% (*n* = 224)	Statistics
	Mean (SD)	Mean (SD)	
Epigenetic age (years)	57.9 (10.2)	55.7 (11.6)	*t* = 2.31, *p* = 0.021
Epigenetic age acceleration	−1.72 (8.13)	0.56 (8.14)	*t* = −3.29, *p* = 0.001
	% (*n*)	% (*n*)	
Epigenetic age acceleration type			*χ* ^2^ = 10.94, *p* = 0.004
Slow ager	16.1 (58)	9.8 (22)	Slow–Fast:
Average ager	76.7 (276)	75.9 (170)	*χ* ^2^ = 9.68, *p*.adj = 0.002
Fast ager	7.2 (26)	14.3 (32)	Other comparisons: NS

Abbreviations: NS, not significant; *P*.adj, Bonferroni adjusted *p* value; SD, standard deviation.

### 3.3 Epigenetic Estimates of Cell‐Type Composition Associated With Morning Fatigue Severity

Higher morning fatigue was associated with lower estimated proportions of CD4 memory, CD8 memory, and NK cell types (Table 4). Higher morning fatigue was associated with higher estimated proportions of neutrophil and T regulatory cell types (Table 4). Morning fatigue severity was not associated with epigenetic estimated proportions of basophile, B‐cell memory, B‐cell naïve, CD4 naïve, CD8 naïve, eosinophil, or monocyte cell types.

**TABLE 4:** Differences in epigenetic estimates of cell type proportions between the morning fatigue severity groups in oncology patients receiving chemotherapy.

Cell type	Description	Low morning fatigue 61.6% (*n* = 360), mean (SD)	High morning fatigue 38.3% (*n* = 224), mean (SD)	Statistics
*Innate immunity*				
Basophils	Granulocyte	0.115 (0.121)	0.125 (0.133)	*t* = 0.92, *p* < 0.358
Eosinophils	Granulocyte	0.005 (0.013)	0.004 (0.012)	*t* = 1.14, *p* < 0.251
Monocytes	Granulocyte	0.101 (0.045)	0.100 (0.047)	*t* = 0.05, *p* < 0.960
Natural killer	NK cells	0.030 (0.019)	0.027 (0.019)	*t* = 1.97, *p* < 0.049
Neutrophils	Neutrophils	0.612 (0.140)	0.637 (0.138)	*t* = 2.10, *p* < 0.036
*Adaptive immunity*				
B memory	B cells	0.018 (0.028)	0.015 (0.009)	*t* = 1.54, *p* < 0.125
B naïve	B cells	0.012 (0.015)	0.012 (0.016)	*t* = 0.08, *p* < 0.934
CD4 memory	T cells	0.073 (0.049)	0.062 (0.043)	*t* = 2.80, *p* < 0.005
CD4 naïve	T cells	0.027 (0.031)	0.028 (0.034)	*t* = 0.44, *p* < 0.657
CD8 memory	T cells	0.056 (0.051)	0.045 (0.043)	*t* = 2.87, *p* < 0.004
CD8 naïve	T cells	0.009 (0.005)	0.009 (0.004)	*t* = 0.23, *p* < 0.815
T regulatory	T cells	0.013 (0.010)	0.016 (0.011)	*t* = 0.03, *p* < 0.002

Abbreviations: CD4, cluster of differentiation 4; CD8, cluster of differentiation 8; NK, natural killer; SD, standard deviation.

### 3.4 Differentially Methylated eCpG Region Associated With Morning Fatigue Severity

Of the four functional categories for eCpG mappings on the same chromosome (i.e., Cis, In gene body, Near promoter, and Distal), only Distal eCpGs had sufficient representation and were evaluated for DMRs (Table S2; (Files S2 and S3), https://doi.org/10.5281/zenodo.14019736). Of the 25,310 Distal eCpG mappings identified in the eQTM mapping analysis (*p* < 1.0 × 10^−6^), 9658 unique CpG loci were identified and evaluated in the DM and DMR analyses. Three surrogate variables were identified and included with the six demographic and clinical characteristics as covariates in the final model. Results of the DM analysis of individual Distal eCpGs are reported in (File S5). The sliding window analysis identified 1285 Distal regions. Results of the DMR analysis of all regions are reported in (File S6). Morning fatigue severity was associated with differential methylation of one region (i.e., Region 15_204, FDR < 0.0002) (Table 5). The five CpG loci of this DMR were mapped to three genes (i.e., CILP1, Cartilage intermediate layer protein 1; ONECUT1, One Cut Homeobox 1; SLCO3A1, Solute Carrier Organic Anion Transporter Family Member 3A1) in the eQTM analysis.

**TABLE 5:** Summary of the differentially methylated distal eCpG region between oncology patients receiving chemotherapy in the low and high morning fatigue groups.

Region	CpG loci	Loci statistics		Region	eCpG mapped genes
Description		logFC	*p* value	Statistics	
15_204	cg03291024	0.168	0.001	*χ* ^2^ = 51.80	CILP
chr15: 91473291—	cg08939371	0.171	0.002	FDR < 0.0002	CILP, ONECUT1, SLCO3A1
91473091	cg16414568	0.139	0.027		CILP, SLCO3A1
	cg18472881	0.178	0.009		CILP, SLCO3A1
	cg18724928	0.174	0.006		CILP, ONECUT1, SLCO3A1

Abbreviations: chr15, chromosome 15; CILP1, cartilage intermediate layer protein 1; eCpG, expression‐associated CpG; FDR, false discovery rate; logFC, log fold‐change; ONECUT1, One Cut Homeobox 1; SLCO3A1, Solute Carrier Organic Anion Transporter Family Member 3A1.

**FIGURE 4:**
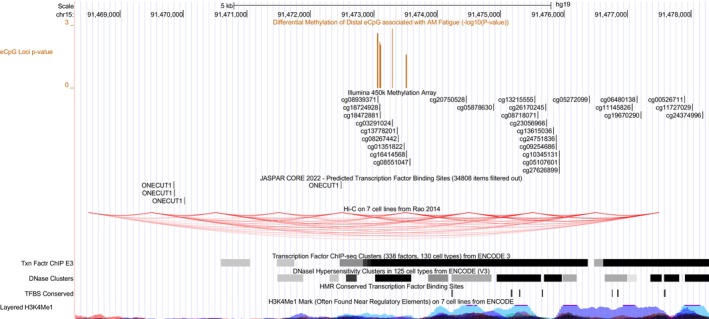
Screenshot of the University of California Santa Cruz Genome browser displaying the region on chromosome 11 of the hg19 (Genome Reference Consortium version 37) assembly of the human genome that includes the region #15_204 identified in this study as differentially methylated and the locations of the methylation probes included in the analysis. Assembly tracks show scale, chromosome, and position of the region. Predicted binding sites for the ONECUT1 transcription factor are shown in the JASPER CORE 2021 track. Putative regulatory regions are identified by the layered H3K4Me1, transcription factor (ChIP‐seq) clusters, and DNase hypersensitivity clusters tracks. Hi‐C track shows heatmaps of chromatin folding data (i.e., interactions detected between regions). ChIP, chromatin immunoprecipitation; H3K4Me, histone H3 lysine 4 methylation.

In terms of a potential functional role, this DMR was located in putative regulatory regions identified by the layered H3K4Me1, transcription factor (ChIP‐seq) clusters, and DNase hypersensitivity cluster tracks (Figure 4). In addition, there was evidence of high interactions (i.e., Hi‐C on 7 cell lines track) of this DMR with nearby predicted transcription factor ONECUT1 binding sites (i.e., JASPER CORE 2021 track). All eCpGs in the region were differentially methylated in the same direction. Higher fatigue was associated with increased methylation.

## 4 Discussion

This systems biology study is the first to provide an epigenetic characterization of morning fatigue severity in oncology patients receiving chemotherapy in terms of EAA, cell‐type compositions, and differential methylation of a regulatory region of distal eCpGs. Each of the individual characterizations will be discussed individually in more detail below. The paper concludes with a brief discussion of these findings in the context of a potential common mechanism (i.e., Inflammaging) and describes opportunities for future research.

### 4.1 Morning Fatigue Severity Is Associated With EAA

Growing evidence suggests EAA is linked to common diseases, morbidity, and mortality and is associated with various social and environmental factors [83, 84]. The difference between chronological and biological age (i.e., EAA) is a valuable biomarker for understanding the risk(s) for common diseases [85]. However, limited studies have explored its utility in understanding symptom burden [86]. A better understanding of an epigenetic role in the aging process is needed to advance the prevention and treatment of aging‐related conditions [87, 88]. Our findings are congruent with previous reports showing an association between EAA and fatigue in oncology patients [15, 32]. This study found that higher EAA is associated with higher morning fatigue, and patients who were “fast” agers were more likely to report higher morning fatigue. These findings support the hypothesis that some patients with higher fatigue are biologically older than their chronological age. Understanding the causes of fatigue in young cancer patients has been challenging, as fatigue symptoms have generally been associated with chronological aging. The associations of EAA with fatigue severity suggest that an older biological age may explain these symptoms in some younger patients. Future studies need to evaluate this hypothesis with other common symptoms (e.g., anxiety, cognitive impairment, sleep disturbance) [24, 89] that are more severe in younger patients.

Although chronological age is a commonly used measure, it does not capture the aging process well [29]. In contrast, biological age is a measure of a person's or organ's functional capability and how it changes with age [27, 28, 29]. Over the past decade, numerous epigenetic age estimators using DNA methylation‐based markers have been developed to reliably predict biological age [90, 91]. Next generation clocks like DNAm PhenoAge were trained to represent mortality risk as well and are often predictive of different health problems. In addition, it demonstrates strong correlations with molecular function (i.e., gene expression) [65, 83]. Given the results of this study and others [15], this clock shows promise for evaluating age‐related changes using estimators of biological age rather than chronological age to help us better understand the biology of aging and fatigue [29]. Future research should evaluate the relationships between the severity of fatigue and other symptoms in the context of biological age, including other measures of epigenetic age [92] and calculations of EAA [93, 94]. In addition, long‐term epigenetic profiling, with longitudinal data, is needed to evaluate the relationships among the aging trajectory, the symptom severity, molecular mechanisms, and other factors (e.g., diet, stress, and trauma) [83, 95].

### 4.2 Morning Fatigue Severity Is Associated With Immune Cell‐Type Compositions

A number of key physiological mechanisms have evolved in mammals to respond to physical damage or other insults (e.g., cancer or its treatment), including the repair and replacement of damaged tissue and the inflammatory response [96, 97]. The blood system is a fundamental contributor to immunity and tissue repair [98]. All blood lineages contribute to the inflammatory response, and these responses are associated with changes in the blood system [97, 98]. A categorization of the blood cell types provides a detailed immune profile. These characterizations provide a basis to better understand the immune profile of morning fatigue in oncology patients.

The inflammatory response can broadly be divided into innate and adaptive immunity. Cells involved in innate immunity are activated in response to acute insults [99]. In the current study, higher levels of morning fatigue were associated with lower NK and higher neutrophil cell proportions. NK cells target and kill aberrant cells (e.g., virally infected and tumorigenic cells) [100]. Killing is mediated by cytotoxic molecules, which are stored within secretory lysosomes. Our findings are consistent with previous work from our group showing that perturbation of the NK cell‐mediated cytotoxicity pathway (KEGG hsa04650) is significantly associated with fatigue severity in patients receiving chemotherapy [33, 36, 37, 101]. However, our findings are not concordant with a previous study of breast cancer survivors where NK T cell counts did not differ between fatigued and matched nonfatigued survivors [102]. These different findings may be due to cancer types, patient characteristics, cell‐type assessment, or fatigue measures. In contrast, in a study of patients with myalgic encephalomyelitis/chronic fatigue syndrome (ME/CFS), NK cells are associated with a diagnosis of ME/CFS and are under exploration as a potential target for intervention [103, 104]. Fatigue and CFS/ME share gene expression patterns at a subset of genes [105] and pathways [33] and may share underlying mechanisms [106, 107].

Neutrophils are the most abundant cell type in blood [108]. Their functions include phagocytosis, degranulation, and the formation of neutrophil extracellular traps (NETs). An increase in a subpopulation of neutrophils (i.e., low‐density granulocytes) is hypothesized to act as NET inducers [109]. Dysregulation of NETs occurs in response to inflammation [101]. Perturbations of the neutrophil extracellular trap formation pathway (KEGG hsa04613) are significantly associated with morning fatigue severity in patients receiving chemotherapy [33]. Our findings are consistent with a study of cancer survivors that found those with chronic fatigue had higher neutrophil counts [110]. Given that NETs are hypothesized to influence factors associated with fatigue (i.e., sleep quality and physical activity) [111, 112], that neutrophil generation and dysregulation may occur in response to acute demands (e.g., cancer or chemotherapy) [99], and that chronic stress is associated with the formation of NETs [113] and with poor survival in cancer patients, further research is needed to evaluate the relationship between NETs and fatigue in oncology patients.

In terms of cells involved in adaptive immunity, adaptive immune cells generally play a larger role in persistent or chronic inflammatory states. In terms of morning fatigue, higher morning fatigue was associated with lower CD4 and CD8 memory cell proportions and higher T regulatory cell proportions. The adaptive immune response of T cells includes activation, differentiation, and memory formation [114]. Naïve T cells are produced in bone marrow and migrate to the thymus and periphery for differentiation [114, 115]. T cells differentiate into numerous cell types, including CD4, CD8, T regulatory, and others [116]. CD4 T cells can differentiate into T regulatory cells. These cells have a specific function in the immune response. After the primary response is over, the majority of the T cells are removed, and a small proportion of memory T cells remain. Memory T cells are continuously derived from their naïve precursors.

CD4 T cells function as “helpers” through cytokine and chemokine production to activate or recruit nearby immune cells [114] while CD8 T cells function as “cytotoxic” by eliminating target cells using cytotoxic processes [114]. Our findings are inconsistent with two studies of breast cancer survivors where CD4+ T cells were lower in fatigued compared to matched nonfatigued survivors [102, 117]. In terms of CD8 T cells, our findings are not concordant with a previous study of 50 breast cancer survivors that found no association between CD8+ T cell levels between fatigued and matched nonfatigued survivors [102]. In contrast, the difference in the proportions of memory cells associated with fatigue severity may reflect a decline in the adaptive immune cell functions. The discordance in CD4+ and CD8+ T cell levels may be due to differences between measures of fatigue (i.e., morning versus mean fatigue), the mechanisms of fatigue in patients versus survivors, the resolution of distinct cell types (e.g., naïve, memory, and regulatory), sample size, or tissue type (i.e., leukocytes or whole blood). Given the utility of the immune characterization in the evaluation of fatigue, the epigenetic regulation of the transition of naïve to memory/effector states in T cells [118, 119], and the additional cell states not evaluated (e.g., effector T cells), additional research is needed to characterize the immune microenvironment (e.g., cytometry by time of flight (CyTOF), single‐cell RNA‐seq, ATAC‐seq) and its relation to fatigue severity.

Finally, T regulatory cells function as mediators by maintaining immune homeostasis [120]. They perform direct and indirect immunosuppressive activity [121]. High regulatory T‐cell activity is associated with autoimmune diseases [122] and the impairment of host tumor surveillance [120]. Given we see higher proportions of T regulatory cells in patients reporting higher morning fatigue and that these cell populations are candidates for targeted interventions [120, 123], evaluations of fatigue severity and T regulatory cell proportions are needed.

### 4.3 Morning Fatigue Severity Is Associated With a Differentially Methylated Distal Region of eCpGs

The evaluation of DNA methylation in enhancer regulatory regions, rather than promoter regions, can provide unique insight into epigenetic regulatory activity associated with morning fatigue that would be otherwise missed by focusing on promoter regions [124]. In this study, we identified one DMR associated with morning fatigue severity, which maps to the expression levels of three genes (i.e., SLCO3A1, CILP1, and ONECUT1).

The Solute Carrier Organic Anion Transporter Family Member 3A1 (SLCO3A1) is predicted to enable sodium‐independent organic anion transmembrane transporter activity. It is involved in positive regulation of nuclear factor kappa‐light‐chain enhancer of activated B‐cell (NF‐κB) transcription factor activity, positive regulation of protein phosphorylation, and prostaglandin transport. In terms of morning fatigue, perturbation of the NF‐κB signaling pathway is significantly associated with morning fatigue severity in patients receiving chemotherapy [33] and is highly interconnected with other perturbed inflammatory pathways. In addition, compared to breast cancer survivors without fatigue, increased signaling in this pathway is observed in breast cancer survivors with persistent fatigue [125]. While associations between genetic variations associated with this gene and fatigue have not been evaluated in patients undergoing chemotherapy, in a genome‐wide association study (GWAS) of patients with ME/CFS, genetic variations in SLCO3A1 (rs8029503, *p* < 5.66 × 10^−11^) were associated with a diagnosis of ME/CFS [126].

The cartilage intermediate layer protein 1 (CILP1) gene encodes for an extracellular matrix protein. CILP1 mRNA expression is induced by the transforming growth factor beta (TGF‐β1) signaling pathway, and the CILP protein binds and inhibits TGF‐β1 [127]. TGF‐β is the major regulator of the profibrotic response across tissues [128]. Through a feedback loop, CILP‐1 protects against persistent TGFβ1 activity and has an antifibrotic effect in pressure overload‐induced fibrotic remodeling [129]. The fibrosis process is associated with chronic inflammation, where extracellular matrix components build up to cause fibrosis [130]. A unique aspect of fibrosis development is the dysregulation of normal healing processes [131]. Of note, in our recent study of patients receiving chemotherapy [33], morning fatigue severity was associated with RNA expression perturbations in the TGF‐β1 signaling pathway (KEGG hsa04350; *χ* ^2^ = 15.02, *p* < 0.005). In addition to the dysfunction of TGF‐β1, age‐related fibrosis is associated with the release of NETs [132]. As described in the previous section, higher morning fatigue is associated with higher levels of neutrophils and perturbations of the neutrophil extracellular trap formation pathway. Given fibrosis can occur in some patients receiving chemotherapy, [131] future research is needed to evaluate for common and distinct molecular mechanisms for morning fatigue and fibrosis.

Finally, the ONECUT1 gene product is a transcription factor that antagonizes glucocorticoid‐stimulated gene transcription and is involved in glucose metabolism and cell cycle regulation. Misregulation of the Onecut (Oc) family members of transcription factors has been identified as an important factor in disease development [133]. The Oc family is associated with the initiation of endocrine differentiation, and ONECUT1 is required to ensure endocrine differentiation [133]. Notably, fatigue has been linked to numerous metabolic and neuroendocrine pathways (See reviews in [5, 8, 19, 134, 135]). Given its critical role in regulating physiological processes, additional research is needed to evaluate for the relationships between the neuroendocrine system, endocrine differentiation, and fatigue [5, 8, 19].

### 4.4 Inflammaging and Fatigue in Oncology Patients as a Potential Common Mechanism

In this study, morning fatigue severity is associated with EAA, numerous innate and adaptive immune cell types, and expression‐associated methylation differences in regulatory regions of genes involved in inflammatory processes. Previous authors have proposed that inflammation is a target to reduce the impact of EAA on poor functional outcomes [15] and that administering anti‐inflammatory drugs during chemotherapy might result in more successful clinical results [136].

A better understanding of inflammation as it relates to these different biomarkers is an important next step to better understand the biological cause of morning fatigue. In terms of the molecular mechanisms associated with EAA, nine age‐related processes have been identified as hallmarks of aging [137]. One of the hallmarks, age‐related decline in the immune system (i.e., Inflammaging) [138, 139], hypothesizes that a continuous antigenic load and stress provokes “a global reduction in the capacity to cope with a variety of stressors and a concomitant progressive increase in proinflammatory status.” [139] Another definition of Inflammaging is a “sterile, non‐resolving, low‐grade, and chronic inflammation that progressively increases with age” [140].

One of the tenets of Inflammaging is that the aging process that manifests later starts early in life [139, 141]. For example, early life stressors are associated with neurodevelopmental conditions [142]. In terms of morning fatigue in patients receiving chemotherapy, latent class analysis was used to identify patient subgroups with morning fatigue profiles and evaluated for differences in stress among the fatigue classes [52]. Compared with other classes, patients in the Very High morning fatigue class reported a higher number of and greater impact from previous stressful life events and higher general stress.

Inflammaging is a multilevel inflammatory process that includes numerous changes in the adaptive and innate immune system associated with age [140, 143]. In terms of the adaptive immune system, findings from the current study identified age‐related changes supporting the inflammaging processes that may reflect a lifetime exposure of an individual to stress (i.e., morning fatigue was associated with fewer memory CD4+ and CD8 T cells) but not thymic involution (i.e., morning fatigue was not associated with fewer naïve CD4+ and CD8+ T cells). The processes of dysfunction of T cells and changes in cell membranes with aging were not evaluated. In terms of the innate immune system, findings from the current study identified age‐related changes associated with fatigue severity. Our previous work [33] supported the dysfunction of numerous signaling pathways associated with response to immune challenges (i.e., morning fatigue is associated with perturbations in Toll‐like receptor signaling, AGE‐RAGE signaling pathway in diabetic complications, and NOD‐like receptor signaling) and dysfunction of phagocytosis (i.e., morning fatigue is associated with perturbations in the Phagosome pathway). Although we found decreases in NK cells and increases in neutrophils were associated with morning fatigue severity, the processes of changes in phenotypes of monocyte and NK cells were not evaluated. Other processes that may contribute to inflammaging include the senescence‐associated phenotype, the development of gut dysbiosis, lifelong stimulation of the immune system, and epigenetic and metabolic changes. Given the overlap between Inflammaging and epigenetic aging is not well understood [144], a more complete evaluation of the relative roles of these processes in fatigue is needed.

## 5 Limitations

Given that this study is the first to report on associations between morning fatigue and EAA, cell‐type proportions, and differential methylation of an express‐associated regulatory region, these findings warrant confirmation. In addition, more studies are needed to evaluate patients receiving other types of treatments (e.g., radiation or combined chemotherapy and radiation). Given the association between EAA and immune cell types [145], future studies are needed to evaluate the relative contributions of EAA and detailed measures of cell‐type compositions (e.g., CyTOF and single‐cell analyses). In addition, given that epigenetic clocks capture different aspects of biological aging [146], future studies are needed to evaluate other epigenetic clocks. Future studies are also needed to evaluate the contributions of these EAA types and cell types on morning fatigue in the context of other covariates. Finally, longitudinal studies are needed to assess the associations between changes in the severity and distress of morning fatigue and these epigenetic processes.

## 6 Conclusions

This is the first study to report the association between morning fatigue severity in patients receiving chemotherapy and EAA, faster EAA aging types, and numerous cell‐type compositions. In addition, this study is also the first to identify eCpGs for the peripheral blood of oncology patients receiving chemotherapy using eQTM analysis and to identify a DMR of distal eCpGs associated with morning fatigue severity. Finally, this study is the first to evaluate the role of Inflammaging processes in morning fatigue. This data‐driven approach has provided an initial epigenetic characterization of morning fatigue in oncology patients receiving chemotherapy and has identified numerous opportunities for future research.

## Author Contributions

**Caroline Le:** writing – original draft, writing – review and editing, formal analysis, data curation, validation, methodology, conceptualization. **Maureen Lewis:** writing – review and editing, formal analysis, data curation. **Carolyn S. Harris:** writing – review and editing. **Liam Berger:** software, writing – review and editing. **Esther Chavez‐Iglesias:** writing – review and editing. **Lisa Morse:** writing – review and editing. **Anatol Sucher:** writing – review and editing. **Ritu Roy:** writing – review and editing, formal analysis, methodology. **Adam Olshen:** writing – review and editing, formal analysis, methodology. **Marilyn J. Hammer:** writing – review and editing. **Steve Paul:** writing – review and editing. **Margaret Wallhagen:** writing – review and editing. **Raymond Chan:** writing – review and editing. **Michael Sayer:** writing – review and editing. **Sue Yom:** writing – review and editing. **Nam‐Woo Cho:** writing – review and editing. **Alexandre Chan:** writing – review and editing. **Jon Levine:** writing – review and editing. **Anand Dhruva:** writing – review and editing. **Christine Miaskowski:** writing – review and editing, resources. **Yvette P. Conley:** writing – review and editing, methodology. **Kord M. Kober:** writing – review and editing, formal analysis, software, conceptualization, methodology, investigation, funding acquisition, writing – original draft, resources, project administration, visualization, supervision, data curation, validation.

## Ethics Statement

The study was approved by the Committee on Human Research at the University of California San Francisco and the Institutional Review Board at each of the study sites.

## Consent

Patients gave written informed consent.

## Conflicts of Interest

The authors declare no conflicts of interest.

## Supporting information


Data S1.

Data S2.

Data S3.

Data S4.

Data S5.

Data S6.

## Data Availability

The data that support the findings of this study are available from the corresponding author upon reasonable request.
