# Supplementary material for: A Data‐Driven Epigenetic Characterization of Morning Fatigue Severity in Oncology Patients Receiving Chemotherapy: Associations With Epigenetic Age Acceleration, Blood Cell Types, and Expression‐Associated Methylation
Source: Cancer Med. 2025 Jul 25;14(15):e71067. doi: 10.1002/cam4.71067 (PMC12290682; doi:10.1002/cam4.71067)
Supplement: Supplementary file 1 — Data S1. [file CAM4-14-e71067-s003.docx]

Supplemental File 1

Expression Quantitative Trait Methylation (eQTM) Mapping Analysis to Identify Expression Associated CpG Loci (eCpGs).

**Acquisition and Processing of Gene Expression and DNA Methylation Data**

Gene expression quality control procedures are described in detail elsewhere.^1^ In brief, total RNA isolated from peripheral blood was quantified for 115 patients using the HumanHT-12 v4.0 Expression BeadChip (Illumina, San Diego, CA) microarray. Gene expression values were log-transformed, background corrected, and normalized using the neqc() function using the limma package.^2^

DNA methylation analyses are described in detail elsewhere.^3^ In brief, total DNA isolated from peripheral blood was quantified for 115 patients using the Infinium HumanMethylation450 BeadChip (Illumina, Inc., San Diego, CA). Corrections for probe type, balance correction, background correction, and quantile normalization were performed using the minfi package (version 1.40.0).^4, 5^ Methylation values were quantified as beta scores for the expression quantitative trait methylation (eQTM) mapping analysis.^6^

**EQTM Mapping**

While evidence supports a relationship between methylation and gene expression, the patterns of these associations can vary.^72^ Gene expression can be associated with decreased^44^ and increased^45^ methylation in regulatory regions and decreased methylation within the gene.^46^ To identify expression-associated CpG (eCpG) loci, eQTM analysis was performed with data from 115 patients for which both gene expression and methylation microarray data were available. The eQTM analysis was performed using the Torch-eCpG tool^7^ (https://github.com/kordk/torch-ecpg) to map eCpGs (GPU accelerated analysis, chuck sizes: n=100 expression probes and n=100,000 methylation loci, filter p<1 x 10^-5^). Chronological age (years) was included in the model as a covariate for the multivariate regression. Putative eCpGs were identified as having an uncorrected p-value < 1 x 10^-6^.^6^ HT-12 microarray probes were annotated for 30,953 genes using the Re-Annotator database.^8^ For probes with multiple annotations, the first gene symbol listed was used. Methylation microarray probes (n=402,147) were annotated using “IlluminaHumanMethylationEPICanno.ilm10b4.hg19” annotation (https://bitbucket.com/kasperdanielhansen/Illumina_EPIC). All eCpGs were categorized into one of five functional regions or as unassigned.^6^ To characterize the function of eCpGs identified in this study, functional enrichment analysis of gene ontology (GO) annotations,^9^ Reactome,^10^ WikiPathways,^11^ and Kyoto Encyclopedia of Genes and Genomes (KEGG) pathways^12^ of the associated transcripts was performed with ToppFun.^13^

**EQTM Mapping in Peripheral Blood from Oncology Patients Receiving Chemotherapy**

Demographic and clinical characteristics of the 115 patients evaluated in the eQTM mapping analysis are provided in Supplemental Table 1. A total of 30,912 gene expression probes and a total of 431,483 methylation probes were included in the analysis. Mapping of the approximately 1.34 x 10^10^ pairwise comparisons completed in approximately one hour using an A2 GPU (Nvidia Corporation, Santa Clara, CA). A total of 84,047 eCpGs were identified. Of the 1,458,147 eQTM mappings identified, 973,944 annotations were identified for both CpG loci and genes and annotated for the eCpG functional category (Supplemental Table 2). All eQTM mapping results for significant eCpGs are available in Supplemental File 2. Functional region assignments for each eCpG are provided in Supplemental File 3. Supplemental Files 2 and 3 are available online at Zenodo (https://doi.org/10.5281/zenodo.14019736).

Transcripts associated with Distal eCpGs were enriched for eight GO molecular functional categories, three KEGG, fourteen Reactome, and six WikiPathway pathways (Supplemental File 4). Pathway enrichment analysis identified numerous mechanisms, including translation (i.e., Ribosome, KEGG M189; Translation initiation, KEGG M47785; Translation initiation, Reactome M27686; Translation elongation, Reactome M29556), metabolism of RNA (i.e., Nonsense mediated decay (NMD), Reactome M27921), cellular development and differentiation (e.g., Developmental biology, Reactome M509; Nervous system development, Reactome M29853; Pluripotent stem cell differentiation, Reactome M39427), cancer (i.e., Pathways in cancer, KEGG M12868), and targets for chemotherapy (i.e., Cytoskeletal motor activity, GO:0003774; Microtubule motor activity, GO:0003777).

**EQTM Mapping of eCpGs in Oncology Patients Receiving Chemotherapy**

ECpGs are useful in understanding the role of epigenetics in gene regulation. Given that eCpGs are tissue- and patient-population-specific,^6^ eCpG datasets that are more closely related to a study design of interest may provide insights unavailable in unrelated tissues or samples. In addition, having an increasingly diverse set of eCpG datasets allows for a more global understanding of the contribution of methylation status to the epigenetic regulation of gene expression. A growing set of eCpGs have been identified across tissues and exposures (i.e., patients with atherosclerosis,^6, 14^ adults experiencing civilian trauma,^6^ children’s blood,^15^ nasal epithelium,^16^ human skeletal muscle,^17^ and tumor cells^18^). This study provides a novel contribution of an eCpG dataset derived from the peripheral blood of oncology patients receiving chemotherapy. The majority of eCpGs were identified in distal and trans regions, supporting previous findings^6^ that eCpGs are not primarily located near the associated genes. Our findings suggest that methylation of CpGs may play a larger role in gene expression regulation at remote enhancer sites rather than promoter-associated sites. Although differential methylation studies often use promoter-associated regions to identify CpG loci for candidate analyses, promoter-associated CpGs exhibit lower variability relative to enhancer sites^14^ and may be more difficult to associate with disease or other outcomes (e.g., morning fatigue). Enhancer-associated eCpGs offer a functional *a priori* hypothesis for selecting CpG loci for analysis other than promoter regions.

The functional characterization of the transcripts associated with eCpGs in breast cancer patients receiving chemotherapy identifies numerous mechanisms, including translation, RNA metabolism, cancer, chemotherapy targets, and cellular development and differentiation. Epigenetic processes are associated with both cancer^19^ and its treatment.^20^ Given the eCpGs were identified in a population of primarily oncology patients with breast cancer, future research should look at other cancer sites, other tissues, other time points in the patient care trajectory (e.g., prior to and following treatment), and in pre-clinical models. In addition, given the scope of this study was to evaluate distal enhancer regions and the growing interest in the regulation of gene expression from trans regions, future research should characterize the trans eCpGs in patients receiving chemotherapy.

Supplemental Table 1. Demographic and Clinical characteristics of oncology patients (n=115) evaluated in the expression quantitative trait methylation (eQTM) analysis to identify expression associated CpGs (eCpGs).

| Characteristics | Mean (SD) |
| --- | --- |
| Age (years) | 52.5 (11.3) |
| Education (years) | 16.3(2.9) |
| Body mass index (kg/m^2^) | 26.8 (6.7) |
| KPS score | 79.3 (11.3) |
| Number of comorbidities | 2.5 (1.4) |
| SCQ score | 5.6 (3.1) |
| AUDIT score | 2.7 (1.9) |
| Time since diagnosis (years) | 3.1 (4.7) |
| Time since diagnosis (years, median) |  |
| Number of prior cancer treatments | 2.1 (1.9) |
| Number of metastatic sites including lymph node involvement | 1.0 (1.3) |
| Number of metastatic sites excluding lymph node involvement | 0.6 (1.1) |
| MAX2 score | 0.2 (0.1) |
| Hemoglobin (g/dL) | 11.5 (1.3) |
| Hematocrit (%) | 34.3 (3.9) |
| LFS Morning Fatigue score at enrollment | 3.6 (2.2) |
|  | % (n) |
| Gender  Female  Male | 100.0 (115)  0 (0) |
| Ethnicity  White  Black  Asian or Pacific Islander  Hispanic mixed or other | 63.5 (73)  7.8 (9)  17.4 (20)  11.3 (13) |
| Married or partnered (% yes) | 70.4 (81) |
| Lives alone (% yes) | 15.7 (18) |
| Childcare responsibilities (% yes) | 32.2 (37) |
| Care of adult responsibilities (% yes) | 12.2 (14) |
| Born prematurely (% yes) | 3.5 (4) |
| Currently employed (% yes) | 33.0 (38) |
| Income  <$30,000  $30,000 to <$70,000  $70,000 to <$100,000  ≥$100,000 | 25.2 (29)  19.1 (22)  14.8 (17)  40.9 (47) |
| Specific comorbidities (% yes)  Heart disease  High blood pressure  Lung disease  Diabetes  Ulcer or stomach disease  Kidney disease  Liver disease  Anemia or blood disease  Depression  Osteoarthritis  Back pain  Rheumatoid arthritis | 1.7 (2)  27.0 (31)  3.5 (4)  8.7 (10)  2.6 (3)  0.9 (1)  6.1 (7)  18.3 (21)  30.4 (35)  15.7 (18)  33.0 (38)  4.3 (5) |
| Exercise on a regular basis (% yes) | 79.1 (91) |
| Smoking current or history of (% yes) | 25.2 (29) |
| Cancer diagnosis  Breast  Gastrointestinal  Gynecological  Lung | 99.1 (114)  0.9 (1)  0 (0)  0 (0) |
| Type of prior cancer treatment  No prior treatment  Only surgery, CTX, or RT  Surgery & CTX, or surgery & RT, or CTX & RT  Surgery & CTX & RT | 20.9 (24)  43.5 (50)  13.0 (15)  22.6 (26) |
| CTX cycle length  14 day cycle  21 day cycle  28 day cycle | 28.7 (33)  62.6 (72)  8.7 (10) |
| Emetogenicity of CTX  Minimal/low  Moderate  High | 29.6 (34)  40.9 (47)  29.6 (34) |
| Antiemetic regimens  None  Steroid alone or serotonin receptor antagonist alone  Serotonin receptor antagonist and steroid  NK-1 receptor antagonist and two other antiemetics | 13.9 (16)  24.3 (28)  38.3 (44)  23.5 (27) |

Abbreviations: AUDIT = Alcohol Use Disorders Identification Test; CTX = chemotherapy; dL = deciliter; g = grams; kg = kilograms; KPS = Karnofsky Performance Status; LFS = Lee Fatigue Scale; m^2^ = meter squared, NK-1 = neurokinin-1; RT = radiation therapy; SCQ = Self-administered Comorbidity Questionnaire; SD = Standard Deviation

Supplemental Table 2. Expression-associated CpG (eCpG) loci (n=84,047) in oncology patients receiving chemotherapy (n=115)

| Functional  Category^1^ | Description | eCpG^2^  Count | Transcript  Count | eCpG-  Transcript  Pair Count |
| --- | --- | --- | --- | --- |
| Cis | CpG position <50Kb upstream of gene transcript start site | 22 | 17 | 22 |
| In gene body | CpG position within gene transcript start and end sites | 0 | 0 | 0 |
| Near promoter | CpG position within +/- 2.5Kb of gene transcript start site | 8 | 7 | 8 |
| Distal | CpG position >50Kb upstream of gene transcript start site | 9,658 | 6,032 | 25,310 |
| Trans | CpG position on a different chromosome from gene | 68,050 | 18,376 | 948,604 |
| Unassigned | Annotation for CpG or gene missing | 6,309 | 8,655 | 484,203 |
| Total |  | 84,047 | 33,087 | 1,458,147 |

^1^Functional categories follow methylation-expression roles in Kennedy et al. 2018

^2^p < 1 x 10^-6^

Abbreviations: bp, base pair; CpG, cytosine and guanine bases connected by a phosphate group from base pairing; Kb, kilobase

References

1. Singh KP, Dhruva A, Flowers E, Paul SM, Hammer MJ, Wright F, Cartwright F, Conley YP, Melisko M, Levine JD, Miaskowski C, Kober KM. Alterations in Patterns of Gene Expression and Perturbed Pathways in the Gut-Brain Axis Are Associated With Chemotherapy-Induced Nausea. J Pain Symptom Manage. 2020;59(6):1248-59 e5. Epub 2020/01/11. doi: 10.1016/j.jpainsymman.2019.12.352. PubMed PMID: 31923555; PMCID: PMC7239734.

2. Smyth G. Limma: linear models for microarray data. In: Gentleman R, Carey V, Dudoit S, Irizarry R, Huber W, editors. Bioinformatics and Computational Biology. New York: Springer; 2005. p. 397-420.

3. Kober KM, Lee MC, Olshen A, Conley YP, Sirota M, Keiser M, Hammer MJ, Abrams G, Schumacher M, Levine JD, Miaskowski C. Differential methylation and expression of genes in the hypoxia-inducible factor 1 signaling pathway are associated with paclitaxel-induced peripheral neuropathy in breast cancer survivors and with preclinical models of chemotherapy-induced neuropathic pain. Molecular Pain. 2020;16:1-15. Epub 2020/06/27. doi: 10.1177/1744806920936502. PubMed PMID: 32586194; PMCID: PMC7322824.

4. Aryee MJ, Jaffe AE, Corrada-Bravo H, Ladd-Acosta C, Feinberg AP, Hansen KD, Irizarry RA. Minfi: a flexible and comprehensive Bioconductor package for the analysis of Infinium DNA methylation microarrays. Bioinformatics. 2014;30(10):1363-9. doi: 10.1093/bioinformatics/btu049. PubMed PMID: 24478339; PMCID: 4016708.

5. Du P, Kibbe WA, Lin SM. lumi: a pipeline for processing Illumina microarray. Bioinformatics. 2008;24(13):1547-8. Epub 2008/05/10. doi: 10.1093/bioinformatics/btn224. PubMed PMID: 18467348.

6. Kennedy EM, Goehring GN, Nichols MH, Robins C, Mehta D, Klengel T, Eskin E, Smith AK, Conneely KN. An integrated -omics analysis of the epigenetic landscape of gene expression in human blood cells. BMC Genomics. 2018;19(1):476. Epub 2018/06/20. doi: 10.1186/s12864-018-4842-3. PubMed PMID: 29914364; PMCID: PMC6006777.

7. Kober KM, Berger L, Roy R, Olshen A. Torch-eCpG: a fast and scalable eQTM mapper for thousands of molecular phenotypes with graphical processing units. BMC Bioinformatics. 2024;25(1):71. doi: 10.1186/s12859-024-05670-4.

8. Arloth J, Bader DM, Roh S, Altmann A. Re-Annotator: Annotation Pipeline for Microarray Probe Sequences. PLoS One. 2015;10(10):e0139516. Epub 20151001. doi: 10.1371/journal.pone.0139516. PubMed PMID: 26426330; PMCID: PMC4591122.

9. The Gene Ontology C. The Gene Ontology Resource: 20 years and still GOing strong. Nucleic Acids Res. 2019;47(D1):D330-D8. Epub 2018/11/06. doi: 10.1093/nar/gky1055. PubMed PMID: 30395331; PMCID: PMC6323945.

10. Joshi-Tope G, Gillespie M, Vastrik I, D'Eustachio P, Schmidt E, de Bono B, Jassal B, Gopinath GR, Wu GR, Matthews L, Lewis S, Birney E, Stein L. Reactome: a knowledgebase of biological pathways. Nucleic Acids Res. 2005;33(Database issue):D428-32. doi: 10.1093/nar/gki072. PubMed PMID: 15608231; PMCID: PMC540026.

11. Pico AR, Kelder T, van Iersel MP, Hanspers K, Conklin BR, Evelo C. WikiPathways: pathway editing for the people. PLoS Biol. 2008;6(7):e184. doi: 10.1371/journal.pbio.0060184. PubMed PMID: 18651794; PMCID: PMC2475545.

12. Aoki-Kinoshita KF, Kanehisa M. Gene annotation and pathway mapping in KEGG. Methods in molecular biology (Clifton, NJ. 2007;396:71-91. doi: 1-59745-515-6:71 [pii]. PubMed PMID: 18025687.

13. Chen J, Bardes EE, Aronow BJ, Jegga AG. ToppGene Suite for gene list enrichment analysis and candidate gene prioritization. Nucleic Acids Res. 2009;37(Web Server issue):W305-11. doi: 10.1093/nar/gkp427. PubMed PMID: 19465376; PMCID: PMC2703978.

14. Liu Y, Ding J, Reynolds LM, Lohman K, Register TC, De La Fuente A, Howard TD, Hawkins GA, Cui W, Morris J, Smith SG, Barr RG, Kaufman JD, Burke GL, Post W, Shea S, McCall CE, Siscovick D, Jacobs DR, Jr., Tracy RP, Herrington DM, Hoeschele I. Methylomics of gene expression in human monocytes. Hum Mol Genet. 2013;22(24):5065-74. Epub 20130729. doi: 10.1093/hmg/ddt356. PubMed PMID: 23900078; PMCID: PMC3836482.

15. Ruiz-Arenas C, Hernandez-Ferrer C, Vives-Usano M, Mari S, Quintela I, Mason D, Cadiou S, Casas M, Andrusaityte S, Gutzkow KB, Vafeiadi M, Wright J, Lepeule J, Grazuleviciene R, Chatzi L, Carracedo A, Estivill X, Marti E, Escaramis G, Vrijheid M, Gonzalez JR, Bustamante M. Identification of autosomal cis expression quantitative trait methylation (cis eQTMs) in children's blood. Elife. 2022;11. Epub 20220318. doi: 10.7554/eLife.65310. PubMed PMID: 35302492; PMCID: PMC8933004.

16. Kim S, Forno E, Zhang R, Park HJ, Xu Z, Yan Q, Boutaoui N, Acosta-Perez E, Canino G, Chen W, Celedon JC. Expression Quantitative Trait Methylation Analysis Reveals Methylomic Associations With Gene Expression in Childhood Asthma. Chest. 2020;158(5):1841-56. Epub 20200620. doi: 10.1016/j.chest.2020.05.601. PubMed PMID: 32569636; PMCID: PMC7674990.

17. Taylor DL, Jackson AU, Narisu N, Hemani G, Erdos MR, Chines PS, Swift A, Idol J, Didion JP, Welch RP, Kinnunen L, Saramies J, Lakka TA, Laakso M, Tuomilehto J, Parker SCJ, Koistinen HA, Davey Smith G, Boehnke M, Scott LJ, Birney E, Collins FS. Integrative analysis of gene expression, DNA methylation, physiological traits, and genetic variation in human skeletal muscle. Proc Natl Acad Sci U S A. 2019;116(22):10883-8. Epub 20190510. doi: 10.1073/pnas.1814263116. PubMed PMID: 31076557; PMCID: PMC6561151.

18. Yu X, Cen L, Chen YA, Markowitz J, Shaw TI, Tsai KY, Conejo-Garcia JR, Wang X. Tumor Expression Quantitative Trait Methylation Screening Reveals Distinct CpG Panels for Deconvolving Cancer Immune Signatures. Cancer Res. 2022;82(9):1724-35. doi: 10.1158/0008-5472.CAN-21-3113. PubMed PMID: 35176128; PMCID: PMC9064917.

19. Darwiche N. Epigenetic mechanisms and the hallmarks of cancer: an intimate affair. Am J Cancer Res. 2020;10(7):1954-78. Epub 20200701. PubMed PMID: 32774995; PMCID: PMC7407342.

20. Bates SE. Epigenetic Therapies for Cancer. N Engl J Med. 2020;383(7):650-63. doi: 10.1056/NEJMra1805035. PubMed PMID: 32786190.
